# Plasma Adiponectin Level and Myocardial Infarction: the JMS Cohort Study

**DOI:** 10.2188/jea.JE20080057

**Published:** 2009-03-19

**Authors:** Yu Hatano, Masatoshi Matsumoto, Shizukiyo Ishikawa, Eiji Kajii

**Affiliations:** 1 Sixth-year medical student, Jichi Medical University, Shimotsuke, Tochigi, Japan; 2 Division of Community and Family Medicine, Centre for Community Medicine, Jichi Medical University, Shimotsuke, Tochigi, Japan

**Keywords:** adiponectin, myocardial infarction, atherosclerosis, prospective studies, Japan

## Abstract

**Background:**

Adiponectin is associated with many cardiovascular risk factors. Thus, a relation between adiponectin and subsequent coronary heart disease has been hypothesized. However, the results of prospective studies have been conflicting.

**Methods:**

In this nested case-control study, blood samples were collected from 5243 of 12 490 community residents enrolled in the Jichi Medical School Cohort Study. The samples were taken between 1992 and 1995 and stored until 2007, at which point the plasma adiponectin level was measured.

**Results:**

During an average of 9.4 years of follow-up, 38 patients with myocardial infarction and 89 controls matched for age, sex, and community were identified. Plasma adiponectin concentration did not significantly differ between cases and controls (geometric mean 7.6 [interquartile range, 5.0–12.2] versus 7.4 [5.4–11.0] mg/L, respectively, *P* = 0.57). The odds of myocardial infarction in the lowest tertile of adiponectin concentration was not significantly different from that in the highest tertile, after adjustment for age and sex (OR 1.33; 95% CI, 0.50–3.55) or after further adjustment for other cardiovascular risk factors (OR 1.68; 95% CI, 0.45–6.25). Similarly, there was no significant difference in odds of myocardial infarction between the lowest and highest quartiles of adiponectin concentration.

**Conclusion:**

The results do not support an association between hypoadiponectinemia and myocardial infarction.

## INTRODUCTION

Adiponectin is a 244-amino acid, approximately 30-kDa plasma protein, that is the most abundant gene product from adipose tissue.^1^ Hypoadiponectinemia has been associated, both in animal models and in humans, with various risk factors for atherosclerosis, including hypertension, type 2 diabetes, increased insulin resistance, high triglyceride levels, a low level of high-density (HDL) lipoprotein cholesterol, and inflammation.^2^^–^^10^ Replenishment of adiponectin protects against atherosclerosis in vivo.^11^ It has therefore been hypothesized that adiponectin plays a substantial role in the development of atherosclerosis and subsequent cardiovascular disease in human populations.

Many cross-sectional studies have demonstrated that hypoadiponectinemia is indeed associated with the prevalence of coronary heart disease.^2^^,^^12^^–^^19^ However, because a commercially available method for measuring adiponectin has only recently been introduced, there are still a limited number of prospective studies of the longitudinal relationship between adiponectin level and the incidence of myocardial infarction. Moreover, the results obtained from such studies are conflicting. Some prospective studies suggested a pathogenic role for hypoadiponectinemia in the mortality and incidence of myocardial infarction.^6^^,^^7^^,^^19^ Others have observed no such association,^8^^,^^9^^,^^20^ and have instead indicated that the putative association between adiponectin and myocardial infarction was due to confounding by other cardiovascular risk factors, particularly lipids and the presence of type 2 diabetes, and was not significant when adjusted for these factors.^9^^,^^20^ A recent meta-analysis of 7 prospective studies comprising 1318 patients with coronary heart disease also found no significant association between adiponectin level and coronary heart disease.^21^

Some hospital-based case-control studies have explored the association between plasma adiponectin level and myocardial infarction in Asian populations.^2^^,^^5^^,^^12^^,^^14^^,^^22^ However, there have been no community-based prospective studies evaluating the association in Asia. Consequently, we conducted a nested case-control study using data from the Jichi Medical School (JMS) Cohort Study to examine the association between plasma adiponectin concentration and the risk for myocardial infarction over a 9-year follow-up period.

## METHODS

### Study population

Data collection for the JMS Cohort Study was initiated in 1992. The primary objective of the study was to clarify the relations between potential risk factors and cardiovascular diseases in 12 rural districts in Japan.^23^^–^^29^ The baseline data of this cohort study were obtained between April 1992 and July 1995. If several sets of data were obtained for a single participant during that period, the first set was used as the baseline in the present analysis. Baseline data were collected as part of a national mass-screening program. In Japan, mass screening for cardiovascular diseases has been conducted since 1982, in accordance with the Health and Medical Service for the Aged Act of 1981. Local government offices in each community issued invitations to eligible residents for the mass screening, and personal invitations were also sent to all potential participants by mail. As a result, 12 490 participants were eligible (4913 men and 7577 women) across all ages (19 to 93 years). Among the participants, 65 (0.5%) had a previous history of myocardial infarction. The overall response rate among the 12 communities was 65%. Written informed consent to participate in the study was obtained individually from all participants in the mass screening.

Among the 12 490 participants, 5243 (42.0%) with blood samples in storage in 2007 were included as potential study participants; adiponectin was not measured for the baseline analyses. Among these potential participants, 252 individuals with at least 1 cardiovascular event (stroke or myocardial infarction) and 756 matched controls were extracted (a case to control ratio of 1 to 3). Controls were matched for age (using 5-year age increments), sex, and community. Of the 1008 participants recruited, we excluded 38 whose blood sample volume was insufficient for adiponectin measurement, 9 who refused to participate in the study, and 56 with a past history of myocardial infarction or stroke. Among the remaining 905 participants, 38 cases with myocardial infarction and 89 controls were analyzed as the participants in this study. Participants with stroke and their matched controls were analyzed in another study.^30^

### Measurement of baseline variables

To synchronize data collection methods, we established a central committee composed of the chief medical officers of all 12 participating districts. This committee developed a detailed manual for data collection. Body weight was recorded with the participant clothed, and 0.5 kg in summer or 1 kg during the other seasons was subtracted from the recorded weight. Body mass index (BMI) was calculated as weight (kg)/height^2^ (m^2^). Systolic blood pressure and diastolic blood pressure were measured with a fully-automated sphygmomanometer, the BP203RV-II (Nippon Colin), placed on the right arm of a seated participant who had rested in a sitting position for 5 minutes before measurement. Information about medical history and lifestyle was gathered by means of a written questionnaire.

Blood samples were drawn from the antecubital vein of seated participants, with minimal tourniquet use. Specimens were collected in siliconized vacuum glass tubes containing a 1/10 volume of 3.8% trisodium citrate for measurement of blood glucose, and no additives for lipids. Tubes were centrifuged at 3000g for 15 minutes at room temperature. After separation, the serum samples were stored at 4 °C in refrigerated containers if analysis was to be performed within a few days. Otherwise, the samples were frozen until analysis. Plasma samples were frozen as rapidly as possible to −80 °C for storage until laboratory analysis could be performed.

Total cholesterol and triglycerides were measured using an enzymatic method (Wako; inter-assay coefficient of variation [CV]: 1.5% for total cholesterol and 1.7% for triglyceride). HDL cholesterol was measured using the phosphotungstate precipitation method (Wako; inter-assay CV: 1.9%). Blood glucose was measured via an enzymatic method (Kanto Chemistry; inter-assay CV: 1.9%). High-sensitivity C-reactive protein (CRP) level was measured using nephelometry—a latex particle- enhanced immunoassay (NA Latex CRP Kit, Dade Behring). The value in the calibrator was assigned from the Certified Reference Material 470 (IRMM), an international plasma protein reference manual. Its inter-assay and intra-assay CVs were 1.18 and 1.36%, respectively; the assay is sensitive enough to detect 0.03 mg of CRP. Plasma adiponectin concentration was measured by solid phase enzyme-linked immunosorbent assay (adiponectin ELIZA kit; Otsuka Pharmaceutical Co., Ltd.) with an inter-assay CV less than 10%. The ideal measurement range was between 0.375 and 12.0 mg/dL, and the threshold of detection was >23.4 pg/mL. Both high-sensitivity CRP and adiponectin were measured in 2007. The other biochemical markers were measured at the time of sample collection.

In this study, blood samples were drawn from 93 (73.2%) participants after an overnight fast. Participants with diabetes were defined as those with currently-treated diabetes, plasma glucose ≥126 mg/dL after an overnight fast, or postprandial blood glucose ≥200 mg/dL. Participants with impaired glucose metabolism were defined as those with currently-treated diabetes, plasma glucose ≥110 mg/dL after an overnight fast, or postprandial blood glucose ≥140 mg/dL.

### Participant follow-up

As part of the national mass-screening program, repeat examinations were used to follow most participants every year. Those examined were asked whether they had experienced a myocardial infarction after enrolling. Participants who did not come to an appointed screening examination were contacted by mail or phone. Public health nurses visited the participants to obtain pertinent information, when necessary. In this study, 100% of the participants were contacted. Those with a history of myocardial infarction were asked where (at which hospital) they had been treated, and the date of medical diagnosis. Medical records at hospitals in the study area were also consulted to determine if these participants had been treated. If an incident was suspected, pertinent electrocardiography was obtained, in accordance with the protocol for diagnostic identification of myocardial infarction. Diagnoses were determined independently, by means of a diagnosis committee composed of 1 radiologist, 1 neurologist, and 2 cardiologists. A diagnosis of myocardial infarction was determined using the criteria of the World Health Organization Multinational Monitoring of Trends and Determinants in Cardiovascular Disease (MONICA) Project—a multinational collaborative project to monitor coronary events from the mid-1980s to the mid-1990s.^31^ Participants who met the MONICA criteria for a nonfatal or fatal “definite myocardial infarction” or “possible myocardial infarction” were defined as myocardial infarction cases.

### Statistical analysis

Statistical analyses were carried out using SPSS for Windows, version 11.5 (SPSS Inc., Japan). Continuous variables were compared between cases and controls using the unpaired *t* test. Categorical variables were compared using the chi-squared test or the Fisher exact test. Correlations between plasma adiponectin levels and selected cardiovascular risk factors were evaluated by estimating age- and sex-adjusted partial correlation coefficients for continuous variables, and by performing the unpaired *t* test for categorical variables.

The association between adiponectin and incident myocardial infarction was examined by means of logistic regression analysis. Goodness-of-fit was confirmed by the Hosmer-Lemeshow method. For logistic regression analysis, adiponectin levels were categorized into tertiles and quartiles, using results from all study participants. Two separate models were generated for regression analysis: Model 1 was adjusted for age and sex, and Model 2 was adjusted for age, sex, BMI, HDL cholesterol, triglyceride, diabetes, smoking status, systolic blood pressure, and high-sensitivity CRP. Log_10_-transformed adiponectin level and the standard deviation of the increase in log_10_ adiponectin were used to test for linear trends across categories.

Levels of adiponectin, triglyceride, and high-sensitivity CRP were not normally distributed; consequently, they were log_10_-transformed in all analyses. All analyses were two-tailed. *P* < 0.05 was considered statistically significant.

### RESULTS

The mean follow-up period was 9.4 years: 6.3 years (SD, 3.5) in cases and 10.7 ± 2.6 years in controls. Adiponectin and high-sensitivity CRP were measured 14.5 ± 0.9 years and 14.3 ± 1.0 years after initial sample collection in cases and controls, respectively.

The characteristics of the cases and controls are shown in Table 1. The proportion of participants with treated hypertension was significantly greater among the cases than controls (28.9% versus 13.5%; *P* = 0.02). Cases also had higher mean systolic blood pressure (146.2 ± 22.8 versus 132.1 ± 22.0 mm Hg; *P* < 0.01) and mean diastolic blood pressure (87.5 ± 14.0 versus 79.4 ± 13.3 mm Hg; *P* < 0.01). Plasma adiponectin concentration was not significantly different between cases and controls (geometric mean 7.6 [inter-quartile range, 5.0–12.2] versus 7.4 [5.4–11.0] mg/L, *P* = 0.57).

**Table 1.** Baseline characteristics of cases with myocardial infarction and matched controls*

	Cases	(*n* = 38)	Controls	(*n* = 89)	*P* value
Age, mean (SD), y	64.8	(8.4)	64.9	(8.4)	0.96
Male sex	29	(76.3)	70	(78.7)	0.77
Body mass index, mean (SD), kg/m^2^	22.6	(3.5)	22.4	(2.5)	0.74
Treated hypertension	11	(28.9)	12	(13.5)	**0.02**
Treated hyperlipidemia	1	(2.6)	3	(3.4)	0.71
Current smoker	14	(36.8)	35	(39.3)	0.47
Current drinker	18	(47.4)	56	(62.9)	0.26
Diabetes^†^	1	(2.6)	6	(6.7)	0.32
Impaired glucose metabolism^‡^	8	(21.1)	9	(10.1)	0.09
Systolic blood pressure, mean (SD), mm Hg	146.2	(22.8)	132.1	(22.0)	**<0.01**
Diastolic blood pressure, mean (SD), mm Hg	87.5	(14.0)	79.4	(13.3)	**<0.01**
Total cholesterol, mean (SD), mg/dl	202.2	(32.8)	190.1	(37.4)	0.09
High density lipoprotein cholesterol, mean (SD), mg/dl	49.1	(12.9)	48.5	(11.1)	0.79
Triglyceride, geometric mean (IQR), mg/dl^§^	96.4	(66.2–147.7)	94.6	(63.5–137.0)	0.85
Adiponectin, geometric mean (IQR), mg/l^§^	7.6	(5.0–12.2)	7.4	(5.4–11.0)	0.57
High-sensitivity C-reactive protein, geometric mean (IQR), ng/ml^§^	629.1	(244.2–1477.2)	542.5	(234.5–1015.0)	0.83

*Data are expressed as number (%) unless otherwise indicated.

^†^Fasting plasma glucose ≥126 mg/dl, postprandial glucose ≥200, or history of treated diabetes.

^‡^Fasting plasma glucose ≥110 mg/dl, postprandial glucose ≥140, or history of treated diabetes.

§Data were log_10_-transformed for analysis.

Abbreviations: SD, standard deviation; IQR, interquartile range

Associations between adiponectin and selected cardiovascular risk factors are shown in Table 2. Among cases, adiponectin levels were negatively correlated with BMI, and positively correlated with HDL cholesterol. Among controls, adiponectin levels were negatively correlated with BMI and triglyceride, and were lower in men and current smokers.

**Table 2.** Age and sex-adjusted associations between plasma adiponectin level and selected cardiovascular risk factors in myocardial infarction cases and matched controls^*^

Risk factor	Cases (*n* = 38)		Controls (*n* = 89)	
	Correlation	*P* value	Correlation	*P* value
	
Body mass index	−0.50	**<0.01**	−0.37	**<0.01**
Systolic blood pressure	−0.18	0.36	−0.02	0.89
Diastolic blood pressure	−0.08	0.69	0.03	0.78
Total cholesterol	0.12	0.50	−0.19	0.08
High density lipoprotein cholesterol	0.35	**0.04**	0.21	0.05
Triglyceride^†^	−0.29	0.08	−0.30	**<0.01**
High-sensitivity C-reactive protein^†^	−0.24	0.16	−0.15	0.15
	
	Geometric mean	*P* value	Geometric mean	*P* value
	
Sex				
Male	7.52		6.55	
Female	7.78	0.88	11.67	**<0.01**
Treated hypertension				
Yes	7.73		9.95	
No	7.55	0.93	7.13	0.08
Current smoker				
Yes	6.93		5.79	
No	8.37	0.34	8.94	**<0.01**
Current drinker				
Yes	7.45		6.99	
No	8.03	0.71	8.39	0.16
Diabetes^‡^				
Yes	5.20		6.62	
No	7.66	0.50	7.47	0.61
Any abnormality of glucose metabolism^§^				
Yes	6.96		7.05	
No	7.76	0.63	7.45	0.78

*Values shown are Pearson partial correlation coefficients for continuous variables and adjusted geometric means for categorical variables.

^†^Adiponectin, triglyceride, and high-sensitivity C-reactive protein were log_10_-transformed for analysis.

^‡^Fasting plasma glucose ≥126 mg/dl, postplandial glucose ≥200, or history of treated diabetes

^§^Fasting plasma glucose ≥110 mg/dl, postplandial glucose ≥140, or history of treated diabetes

Table 3 shows multivariable-adjusted odds ratios for myocardial infarction, by adiponectin level tertile. In Model 1, the odds of myocardial infarction in the lowest adiponectin tertile (Tertile 1) was higher than that in the highest tertile (Tertile 3), but the difference was not statistically significant (Odds ratio [OR] 1.33; 95% CI, 0.50–3.55). In addition, no significant difference was seen in the risk for myocardial infarction between the lowest and highest adiponectin level tertiles in Model 2, after adjustment for several cardiovascular risk factors (OR 1.68; 95% CI, 0.45–6.25).

**Table 3.** Multivariable-adjusted odds ratios for myocardial infarction by adiponectin level tertile (38 cases and 89 controls)

	Range, mg/liter	Cases	Controls	Model 1*			Model 2^†^		
	
				OR	95% CI	*P* value	OR	95% CI	*P* value
Tertile 1	<5.93	14	28	1.33	0.50–3.55	0.57	1.68	0.45–6.25	0.44
Tertile 2	5.93–9.63	12	31	1.01	0.38–2.65	0.99	1.32	0.42–4.18	0.63
Tertile 3	9.63<	12	30	1.00			1.00		

^*^Adjusted for age and sex

^†^Model 1 plus HDL cholesterol, triglyceride, BMI, diabetes, smoking status, systolic blood pressure, high-sensitivity CRP

Table 4 shows multivariable-adjusted odds ratios of myocardial infarction, by adiponectin level quartile. No significant difference was seen in the risk of myocardial infarction between the lowest and highest adiponectin quartiles, either in Model 1 or in Model 2. There was no significant linear association between adiponectin level and the risk for myocardial infarction, when it was entered into logistic regression models as a continuous variable, ie, there was no trend toward a reduced risk of myocardial infarction at higher adiponectin levels (*P* for trend = 0.88 in Model 1, and 0.99 in Model 2).

**Table 4.** Multivariable-adjusted odds ratios for myocardial infarction by adiponectin level quartile (38 cases and 89 controls)

	Range, mg/liter	Cases	Controls	Model 1*			Model 2^†^		
	
				OR	95% CI	*P* value	OR	95% CI	*P* value
Quartile 1	<5.30	11	20	1.12	0.37–3.40	0.84	0.97	0.20–4.69	0.97
Quartile 2	5.30–7.50	9	23	0.78	0.26–2.31	0.65	1.10	0.27–4.42	0.90
Quartile 3	7.50–11.60	7	25	0.55	0.18–1.68	0.29	0.77	0.21–2.88	0.70
Quartile 4	11.60<	11	21	1.00			1.00		

*P* for trend						0.88			0.99
Per standard-deviation increase in log				1.03	0.68–1.59	0.88	1.00	0.55–1.80	0.99

^*^Adjusted for age and sex

^†^Model 1 plus HDL cholesterol, triglyceride, BMI, diabetes, smoking status, systolic blood pressure, high-sensitivity CRP

## DISCUSSION

To our knowledge, this is the first prospective study to assess the independent association between serum adiponectin level and the incidence of myocardial infarction in the Japanese general population. In this study, adiponectin level was not significantly associated with the incidence of myocardial infarction, either when unadjusted or adjusted for conventional cardiovascular risk factors.

Several prospective studies have examined the association between adiponectin level and myocardial infarction. As did experimental and cross-sectional observational studies in the 1990s and early 2000s, the first prospective study—which was nested within the Health Professionals Follow-up Study—showed that plasma adiponectin had a protective role against the incidence and mortality of myocardial infarction; with the lowest quintile used as a reference, the odds ratio of the highest adiponectin quintile was 0.39 (95% CI, 0.23–0.64).^7^ However, the odds ratio increased to 0.56 (95% CI, 0.32–0.99) when adjusted for other cardiovascular risk factors, in particular low- and high-density lipoprotein cholesterol levels.^7^ This study was followed by prospective studies of similar size, which showed no significant association between adiponectin level and the incidence of myocardial infarction.^8^^,^^9^^,^^20^^,^^21^ A meta-analysis of 7 prospective studies comprising 1318 patients with coronary heart disease also showed that the risk for myocardial infarction in the group with adiponectin levels in the highest tertile (odds ratio 0.84; 95% CI, 0.70–1.01) was not lower than that of the lowest tertile group, after adjustment for conventional risk factors.^21^ In a previous study utilizing the same dataset as the present study, we reported that plasma adiponectin level was not predictive of future ischemic stroke or total stroke.^30^ Although the sample size of this previous study (179 cases, 630 controls) was substantially larger than that reported here, and the outcomes involved different disease entities (stroke versus myocardial infarction), the results of both studies are similar in that adiponectin level was not independently associated with atherosclerotic vascular events. Interestingly, an increasing number of studies have indicated that hyperadiponectinemia, not hypoadiponectinemia, may increase the risk of cardiovascular death.^20^^,^^22^^,^^32^^,^^33^ The present study revealed no association between hypoadiponectinemia and myocardial infarction, with or without adjustment for various risk factors. Although the limited sample size makes this finding inconclusive, our result is in accordance with those observed in most previous prospective studies on the association between adiponectin and myocardial infarction, and with our previous study on the association between adiponectin and stroke.

Adiponectin has been causally linked to insulin sensitivity, and possesses anti-inflammatory properties.^34^^,^^35^ Clinically, there is evidence that adiponectin is associated with obesity, type 2 diabetes, metabolic syndrome, dyslipidemia, and hypertension.^2^^–^^5^^,^^10^ In light of this evidence, the absence of an association between plasma adiponectin level and myocardial infarction that was observed in prospective studies, including ours, is surprising. A possible reason for the discrepancy is the multi-functional properties of adiponectin. Adiponectin is not only a marker of atherosclerosis, but also a parameter of systemic inflammation.^36^ The plasma adiponectin level rises in response to systemic inflammation and adiponectin inhibits the inflammatory processes regulating monocyte adhesion, macrophage transformation, and the proliferation of smooth muscle cells in blood vessels.^37^ Hyperadiponectinemia is associated with chronic inflammatory diseases and death due to chronic inflammation.^32^^,^^33^^,^^38^^–^^44^ Low concentrations of plasma adiponectin, which reflect advanced atherosclerosis in patients with myocardial infarction, may be offset or neutralized by the adiponectin increase triggered by systemic inflammation, which often underlies myocardial infarction.

An important limitation of this study is its small sample size: it comprised only 38 cases and 84 controls. It is possible that a significant relationship between adiponectin level and myocardial infarction was overlooked because of this limited sample size. Based on our sample size, the power of a test (1-beta) to detect an odds ratio at 2 with a 5% significance level is 68%. Thus, we cannot completely deny the possibility of an association between adiponectin level and myocardial infarction. Furthermore, in this study the adiponectin concentration was the total of both high-molecular-weight (HMW) and low-molecular-weight (LMW) adiponectin; they were not separated in the analysis. It has been reported that HMW adiponectin is more important than the LMW form, with regard to any protective role against atherosclerosis and coronary artery disease.^45^

Another limitation is the prolonged interval between blood collection and measurement, which might have affected the measurement results. However, past studies in which plasma adiponectin levels have been measured shortly after plasma collection have shown that mean levels of adiponectin in healthy Japanese populations range between 5.9 and 11.4,^2^^,^^14^^,^^46^^–^^48^ a range that accords with the mean adiponectin level detected among the controls in our study (7.4; inter-quartile range, 5.4–11.0). Moreover, in a study using data from the JMS Cohort Study from which our study is nested, Ishikawa et al. observed that plasma C-reactive protein levels from samples stored an average of 13.8 years were highly correlated with those measured immediately after sampling (Pearson's correlation coefficient 0.92; 95% CI, 0.88–0.95). This suggests that plasma proteins (including adiponectin) remain stable over time.

Plasma adiponectin concentration is believed to be a major indicator of systemic atherosclerosis. However, we observed no independent association between adiponectin level and risk for myocardial infarction. However, further studies are needed to confirm the usefulness of adiponectin concentration as a predictor of myocardial infarction in Asian populations.
